# Enhancement Effects of Martentoxin on Glioma BK Channel and BK Channel (α+β1) Subtypes

**DOI:** 10.1371/journal.pone.0015896

**Published:** 2011-03-18

**Authors:** Jie Tao, Jian Shi, Li Yan, Ying Chen, Yan Hong Duan, Pin Ye, Qi Feng, Jian Wei Zhang, Xue Qin Shu, Yong Hua Ji

**Affiliations:** Lab of Neuropharmacology and Neurotoxicology, Shanghai University, Shanghai, People's Republic of China; University of Oldenburg, Germany

## Abstract

**Background:**

BK channels are usually activated by membrane depolarization and cytoplasmic Ca^2+^. Especially,the activity of BK channel (α+β4) can be modulated by martentoxin, a 37 residues peptide, with Ca^2+^-dependent manner. gBK channel (glioma BK channel) and BK channel (α+β1) possessed higher Ca^2+^ sensitivity than other known BK channel subtypes.

**Methodology and Principal Findings:**

The present study investigated the modulatory characteristics of martentoxin on these two BK channel subtypes by electrophysiological recordings, cell proliferation and Ca^2+^ imaging. In the presence of cytoplasmic Ca^2+^, martentoxin could enhance the activities of both gBK and BK channel (α+β1) subtypes in dose-dependent manner with EC_50_ of 46.7 nM and 495 nM respectively, while not shift the steady-state activation of these channels. The enhancement ratio of martentoxin on gBK and BK channel (α+β1) was unrelated to the quantitive change of cytoplasmic Ca^2+^ concentrations though the interaction between martentoxin and BK channel (α+β1) was accelerated under higher cytoplasmic Ca^2+^. The selective BK pore blocker iberiotoxin could fully abolish the enhancement of these two BK subtypes induced by martentoxin, suggesting that the auxiliary β subunit might contribute to the docking for martentoxin. However, in the absence of cytoplasmic Ca^2+^, the activity of gBK channel would be surprisingly inhibited by martentoxin while BK channel (α+β1) couldn't be affected by the toxin.

**Conclusions and Significance:**

Thus, the results shown here provide the novel evidence that martentoxin could increase the two Ca^2+^-hypersensitive BK channel subtypes activities in a new manner and indicate that β subunit of these BK channels plays a vital role in this enhancement by martentoxin.

## Introduction

BK channels (voltage-dependent large-conductance Ca^2+^-activated K^+^ channels),also referred to as Maxi-K channels, resemble a unique class of ion channels that couple intracellular chemical signaling to electric signaling [1]. Though BK channels are activated by both elevated cytosolic Ca^2+^ and membrane depolarization, they can open even in the absence of calcium [2]. Up to present, BK channels have been demonstrated to regulate smooth muscle tone [3], [4], [5], [6], [7], neuronal firing [8], [9], [10], [11], [12], [13], endocrine cell secretion [14], [15], cell proliferation [16], [17] and cell migration [18].

Functional BK channels are a tetramer of pore-forming α subunits encoded by a single gene *Slo* [19], [20] at least. Different from the close homology of voltage-gated K^+^ (Kv) channel, the α subunit of BK channels possesses additional hydrophobic segments including a transmembrane helix (S0) which places the N terminus on the extracellular side of the plasma membrane[21] and a long cytosolic C-terminal (S7-S10) where putative Ca^2+^-binding sites reside[22]. Besides the coexpression of tissue-specific accessory β subunits, alternative splicing of *Slo* gene can also lead to diverse BK channel subtypes with various biophysical and pharmacological properties[23], [24]. Among the BK channel subtypes, some including glioma BK (gBK) and BK channel (α+β1) have been unraveled to share higher Ca^2+^ sensitivity [24], [25], [26]. gBK channel as a novel BK channel isoform is almost exclusively expressed in human glioma cells and the prominent expression is correlated positively with enhanced malignancy grades[27], [28]. The high Ca^2+^ sensitivity of gBK channel appears to be a necessity for the cross-talk between neuregulin-1 receptor erbB2 and the channel[29]. While, BK channel (α+β1) expressed specifically in cardiovascular system is considered a key player in balancing excessive vasoconstriction in virtue of its ability to sense subtle Ca^2+^ change evoked by depolarization[30]. Despite the physiological or pathological importance of the higher Ca^2+^ sensitive BK channels has been clarified to some extent, the structural and molecular mechanism underlying the Ca^2+^ hypersensitivity of the channels still remains unknown.

As is known to all, neurotoxins are invaluable tools for examining structural and functional characteristics of targeting-channels. Martentoxin, a 37 residues toxin from *Buthus martensi* Karsch (BmK), can block BK channel in adrenal medulla chromaffin cells and modulate the activities of neuronal BK channel subtype (α+β4) with Ca^2+^-dependent manner[31], [32]. The neuronal BK channel (α+β4) currents were reduced in the presence of low cytoplasmic Ca^2+^ concentration, but conversely enlarged in the presence of high cytoplasmic Ca^2+^ concentration. Moreover, the interaction between martentoxin and the neuronal BK channel (α+β4) was implicated for a novel drug-docking model. Since gBK and BK channel (α+β1) possessed higher Ca^2+^ sensitivity than the neuronal BK channel (α+β4) in the physiological condition, it is very intriguing to investigate the modulatory characteristics of martentoxin on these two BK channel subtypes.

## Materials and Methods

### Cell culture and transfection

All experiments were performed on the glioma cell lines U251 (World Health Organization grade IV, glioblastoma multiforme) and HEK 293T cell lines. U251 cells and HEK 293T cells were obtained from Shanghai cell bank of Chinese Academy of Science. The cells were both cultured in Dulbecco's modified Eagle medium (DMEM; Life Technologies, Grand Island, NY) supplemented with 10% heat –inactivated fetal bovine serum (FBS; Gibco, Grand Island, NY). Culture dishes were incubated at 37°C in a humidified atmosphere containing 5% CO_2_, and subcultured approximately every 2∼3 days. The plasmids containing hSloα (U23767) and β1 (KCNMB4; U25138) are gifts from N.W. Davies (University of Leicester) and J.D. Lippiat (Leeds university). One day before transfection, HEK 293T cells were transferred to 24-well plates. At 90% confluence, cells were transiently transfected using Lipofectamine2000 (Invitrogen, USA) at a ratio of 2 µL reagent with 1 µg total plasmid per well. Electrophysiological experiments were performed at 1∼2 days after transfection.

### Electrophysiological recordings

Whole-cell voltage-clamp experiments were performed as described previously [33], using an EPC-9 amplifier (HEKA Eletronik, Germany) at room temperature (21°C –25°C). Patch pipettes were fabricated from glass capillary tubes by PC-10 Puller (Narishige, Japan) with the resistance of 2∼3 MΩ. Data acquisition and stimulation protocols were controlled by a Pentium III computer (Legend, Beijing, China) equipped with Pulse/PusleFit 8.3 software (HEKA Eletronik, Germany). Capacitance transients were cancelled. Cells with a seal resistance (Rseal) below 1 GΩ were omitted. Series resistance (Rs) was compensated (75∼80%) to minimize voltage errors, and cells with a uncompensated series resistance (Rs) above 10 MΩ were omitted. Leak subtraction was performed using P/6 protocol. Data were low-passed at 10 kHz. The rate of solution exchange was studied using solutions with different KCl concentrations and found to be about 95% complete within 20 s. For U251 cells, the holding potential was −60 mV. Unless stated specially, all the recordings were done with the pulse of +100 mV. For HEK 293T cells, the holding potential was −80 mV. Unless stated specially, all the recordings were done with the pulse of +80 mV.

### Ca^2+^ fluorescence measurements by Fura-2

The U251 cells were loaded with 12.5 µM fura-2 AM (Molecular Probes, Dojindo Laboratories, Kumamoto, Japan) in this prepared culture medium at 37°C for 1.5 h. The final concentration of 15% Pluronic acid F-127 was<0.02% (wt/vol). The HEK 293T cells were incubated with 5 µM fura-2 AM in HEPES buffered solution for 30 min at 37°C.

The measurement of changes in cellular Ca^2+^ concentration by Fura-2 was performed as described previously[32], [34]. Fluorescence images were acquired with an inverted microscope (IX-70, Olympus Optical Co., Tokyo, Japan) equipped with a digital CCD (charge-coupled device) camera (C4742-95-12NRB, Hamamatsu Photonics K. K., Japan). A high-speed scanning polychromatic light source (C7773, Hamamatsu Photonics K. K., Hamamatsu, Japan) was used for alternating excitations at wavelengths of 340 nm and 380 nm. Data collection and analyses were performed using a Ca^2+^ imaging system (Aquacosmos Ver1.2, Hamamatsu Photonics K. K., Japan). The sampling interval of Fura-2 fluorescence measurements was 5 s.

### Solutions and drugs

In the patch-clamp recordings, the standard bath solution for U251 cells was consisted of the following (in mM): NaCl 150, KCl 5.6, MgCl_2_ 1, CaCl_2_ 2, glucose 10, and HEPES (N-(2-hydroxyethyl) piperazine-N'- (2-ethanesulfonic acid)) acid 10(pH 7.3 with NaOH). Pipette solutions for U251 cells was composed of the following (in mM): KCl 140, MgCl_2_ 1, EGTA 5, HEPES acid 10(pH 7.2 with KOH). The standard bath solution for HEK 293T cells was consisted of the following (in mM): NaCl 135, KCl 5, MgCl_2_ 1.2, CdCl_2_ 2.5, HEPES 5, glucose 10 (pH 7.4 with NaOH). Pipette solutions for HEK 293T cells was composed of the following (in mM): NaCl 10, KCl 117, MgSO_4_ 2, HEPES 10, MgATP 2, EGTA 1 (pH 7.2 with KOH). The total Ca^2+^ to be added to give the desired free concentration was calculated using the program Maxchelator (http://www.stanford.edu/%7Ecpatton/maxc.html).

In the Ca^2+^ imaging, the Krebs buffered solution for U251 cells had a composition of (in mM): NaCl 130, K_2_HPO_4_ 2.4, CaSO_4_1, MgSO_4_ 1, D-glucose 10, and HEPES 10(pH 7.4 with NaOH). The HEPES buffered solution for HEK 293T cells had a composition of (in mM): NaCl 137, KCl 5.9, MgCl_2_ 1,CaCl_2_ 2.5, HEPES 10, glucose 15 (pH 7.4 with NaOH).

The toxin was dissolved in the bath solution, supplemented with 1 mg/mL bovine serum albumin (BSA) in order to prevent adherence of the toxin to the vials and the perfusion apparatus. Application of 1 mg/mL BSA alone did not alter BK channel function. Unless otherwise stated, all reagents were purchased from Sigma.

### Cell Proliferation Assay

Cell proliferation assays were performed by using Cell CountingKit-8 (Dojindo, Kumamoto, Japan). Cells were plated in 96-well plates at 1×10^4^ cells per well and final volume of cell culture medium in each well was 0.2 mL. The second day, medium was removed, and cells were washed once with sterile phosphate-buffered saline (PBS) and treated with serum-free media as “control”. The next day, cells were treated with various concentrations of drugs or chemicals for 48 h. IbTx was added directly to the wells at final concentrations of 100 nM, and the medium was changed daily throughout the course of the experiment. For dose-response experiments, cells were incubated in serum-free media containing 0, 20, 100, 500, and 1000 nM martentoxin. For time course experiments, cell numbers were determined daily. At the indicated time points, the cell numbers in the wells were measured by the absorbance (450 nm) of reduced WST-8 (2-(2-methoxy-4-nitrophenyl)-3-(4-nitrophenyl)-5-(2,4-disulfophenyl)-2H-tetrazolium, monosodium salt). The assay was conducted in five replicate wells for each sample concentration and controls. Three parallel experiments were performed. All experiments that attempted to compare growth of cells under different drug conditions were performed on serum-free culture. The cell viability of samples at each concentration of the drugs or chemicals was calculated by dividing the optical density of samples with that of control.

### Data analysis

Data were analyzed by PulseFit 8.5 (HEKA Eletronik, Germany) and Origin 7.5 (Northampton, Massachusetts, USA). Results of data analysis were expressed as mean±S.E.M. and n represents the number of the cells examined.

The Statistical significance was determined using the unpaired Student's t-Test or one-way ANOVA, and an asterisk denotes P<0.05 unless otherwise stated. The degree of toxin effect was calculated by expressing the remaining current after each drug exposure as a fraction of the current magnitude of the patch prior to the first drug exposure (i.e., fractional current remaining, I_f_).

Dose-response curve for the percent enhancement of gBK or BK channel (α+β1) currents was drawn according to the Hill equation I =  Im/(1+([toxin]/EC_50_)^n^), where Im is maximum enhanced percentage of BK currents, and [toxin] is the concentration of martentoxin. EC_50_ (half-maximal effective concentration) and n denote the toxin concentration of half-maximal effect and the Hill coefficient, respectively.

gBK or BK channel (α+β1) currents was elicited by the step pulses ranging from – 50 to +120 mV for 200 ms with the increments of 10 mV (The holding potentials were held at –60 mV for gBK and −80 mV for BK channel (α+β1), respectively). For determining the voltage dependence of activation, the conductance was calculated using the formula: G(V) =  I(V)/(V–ErK), where I(V) is the currents of gBK or BK channel (α+β1) at the command voltage V, and ErK is the reversal potential. The conductance were normalized to the maximal value and the voltage dependence for activation of gBK and BK channel (α+β1) fitted to a Boltzmann equation: f(x) = –1/(1+exp((x–V_1/2_)/k)) +1, where V_1/2_ is the voltage at which half-maximal activation occurs, and k describes the slope of the fit.

## Results

### Abundance of gBK expression in U251 glioma cells

**Figure 1:**
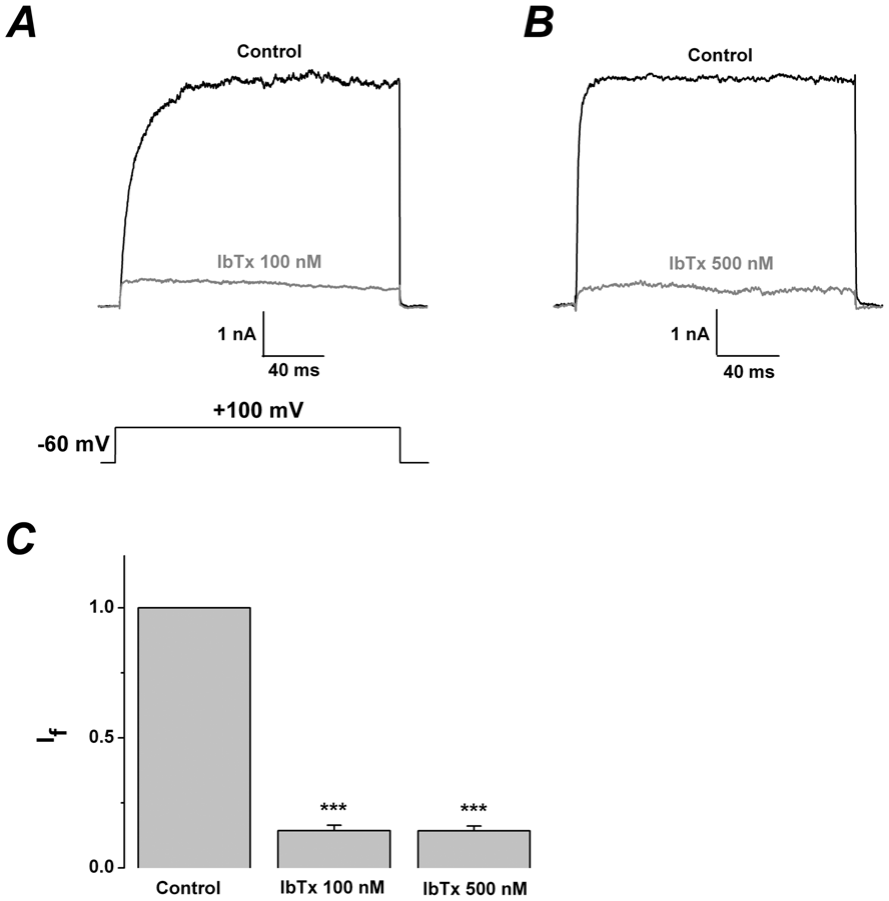
Characterization of gBK expression in U251 cells. (A) Representative whole cell current traces from U251 cells before and after the application of iberiotoxin (IbTx) 100 nM and 500 nM. The holding voltage was −60 mV and the currents were elicited by a pulse of +100 mV with 150 nM free Ca^2+^ in the pipette solution. (B) Statistics analysis of iberiotoxin-sensitive gBK channels in U251 cells as describe in A. Plot of the fraction of unblocked current (I_f_, see “Data analysis”) versus the iberiotoxin concentration. Each point presents data from 6 cells. (n = 6). *** p<0.001.

The outward currents of U251 glioma cells evoked by the pulse of 100 mV were almost completely inhibited by iberiotoxin, a specific BK channel inhibitor. The residual currents in the presence of 100 nM or 500 nM iberiotoxin were 0.14±0.02 (n = 6) or 0.14±0.01 (n = 6) of the control (Fig. 1A and 1B). The abundance of gBK expression in the U251 glioma cells is responsible for ∼90% of the outward currents. The remaining currents could be almost completely inhibited by 200 µM NPPB (5-nitro-2-(3-phenylpropylamino) benzoic acid) (I_f_: 0.021±0.004 compared to 0.14±0.01, p<0.001, n = 6) (Fig. S1). Therefore, the currents are suggested to be NPPB sensitive Cl^−^ channels [28], [35].

### The insensitivity of NPPB sensitive Cl^−^ currents to martentoxin

**Figure 2:**
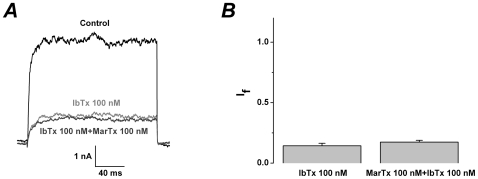
Insensitivity of the remaining iberiotoxin-insensitive outward currents to martentoxin in U251 cells. (A) Representative whole cell current traces from U251 cells before and after the single application of iberiotoxin 100 nM, and simultaneous application of iberiotoxin(IbTx) 100 nM and martentoxin (MarTx) 100 nM. The holding voltage was −60 mV and the currents were elicited by a pulse of +100 mV (Fig. 1A). (B) Statistics analysis of unblocked current (I_f_) after iberiotoxin 100 nM (n = 4) and the mixture of two toxins (n = 4). p>0.05.

As shown in Fig. 1, gBK currents of U251 glioma cells were completely inhibited by 100 nM iberiotoxin. After the pre-treatment of the cells with iberiotoxin (100 nM), the mixture of 100 nM martentoxin and 100 nM iberiotoxin was simultaneously administrated, the remaining outward currents by 100 nM iberiotoxin alone were not significantly different (I_f_: 0.14±0.02 compared to 0.17±0.02, p>0.05,n = 4) (Fig. 2). Obviously, the remaining NPPB sensitive Cl^−^ currents were insensitive to martentoxin.

### Dose-dependent modulation of martentoxin on gBK channels

**Figure 3:**
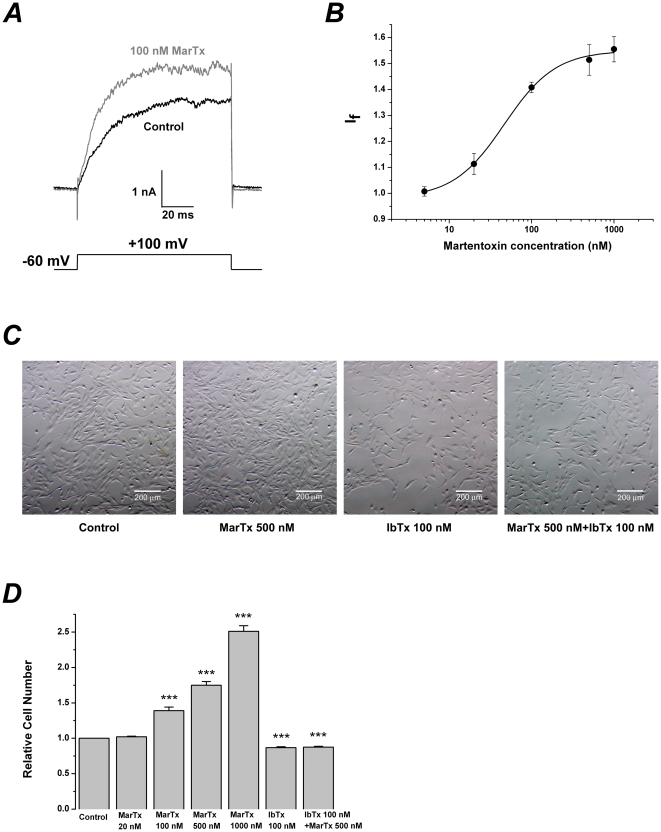
gBK channels were enhanced by martentoxin with dose-dependent manner. (A) Representative whole cell current traces from U251 cells. The holding voltage was −60 mV and the currents were elicited by a pulse of +100 mV. Martentoxin (100 nM) could obviously enhanced the outward currents. (B) The dose-response curve of martentoxin enhancing BK currents was fitted by the Hill equation (see “Data analysis”). The EC_50_ value is 46.7±7.82 nM, and the Hill coefficient is n = 1.40±0.26 (n = 3–8). (C) Images of cells after 2 days under serum-free conditions without toxin (left),with martentoxin (middle), with IbTx or with the mixture of IbTx and martentoxin (right). (D) Relative cell number was measured 2 days after the treatment of variant concentrations of martentoxin (n = 10–15). *** p<0.001.

In the case that the free Ca^2+^ concentration in the pipette solution was controlled to 150 nM, the gBK channel currents could be enhanced by martentoxin in dose-dependent manner. The EC_50_ of martentoxin on gBK channels was assessed to be 46.7±7.82 nM with a Hill coefficient of n = 1.40±0.26 according to the dose-response curve fitting (n = 3–8) (Fig. 3A and 3B).

Free Ca^2+^ concentration in the U251 glioma cells was normally about hundreds nM level and the proliferation of human malignant glioma cells including U251 cells could be decreased by iberiotoxin, a specific gBK channel inhibitor[17]. Unexpectedly, the cell number was significantly increased 48 h after the application of martentoxin under the serum-free cell culture condition. Following the elevated concentration of martentoxin, the cell growth was accelerated likewise. The cell number was increased to 1.02±0.01 (n = 10), 1.39±0.05 (n = 10), 1.75±0.05 (n = 10) and 2.51±0.08 (n = 10) compared with the drug-free controls after the administration of martentoxin 20 nM, 100 nM, 500 nM and 1000 nM, respectively (Fig. 3C and D). On the contrary, the cell number was decreased at 48 h after the application of 100 nM IbTx under the serum-free cell culture condition. Furthermore, the cell number with the treatment of the mixture of 500 nM martentoxin and 100 nM iberiotoxin was not significantly different from that with the treatment of 100 nM iberiotoxin alone (I_f_: 0.87±0.01 compared to 0.86±0.01, p>0.05, n = 15) (Fig. 3C and 3D). This result showed that IbTx could completely abolish the cell proliferation induced by martentoxin, which strongly implied that gBK channel was the unique target of martentoxin.

### Ca^2+^-dependent and voltage-independent modulation of martentoxin on gBK channels

**Figure 4:**
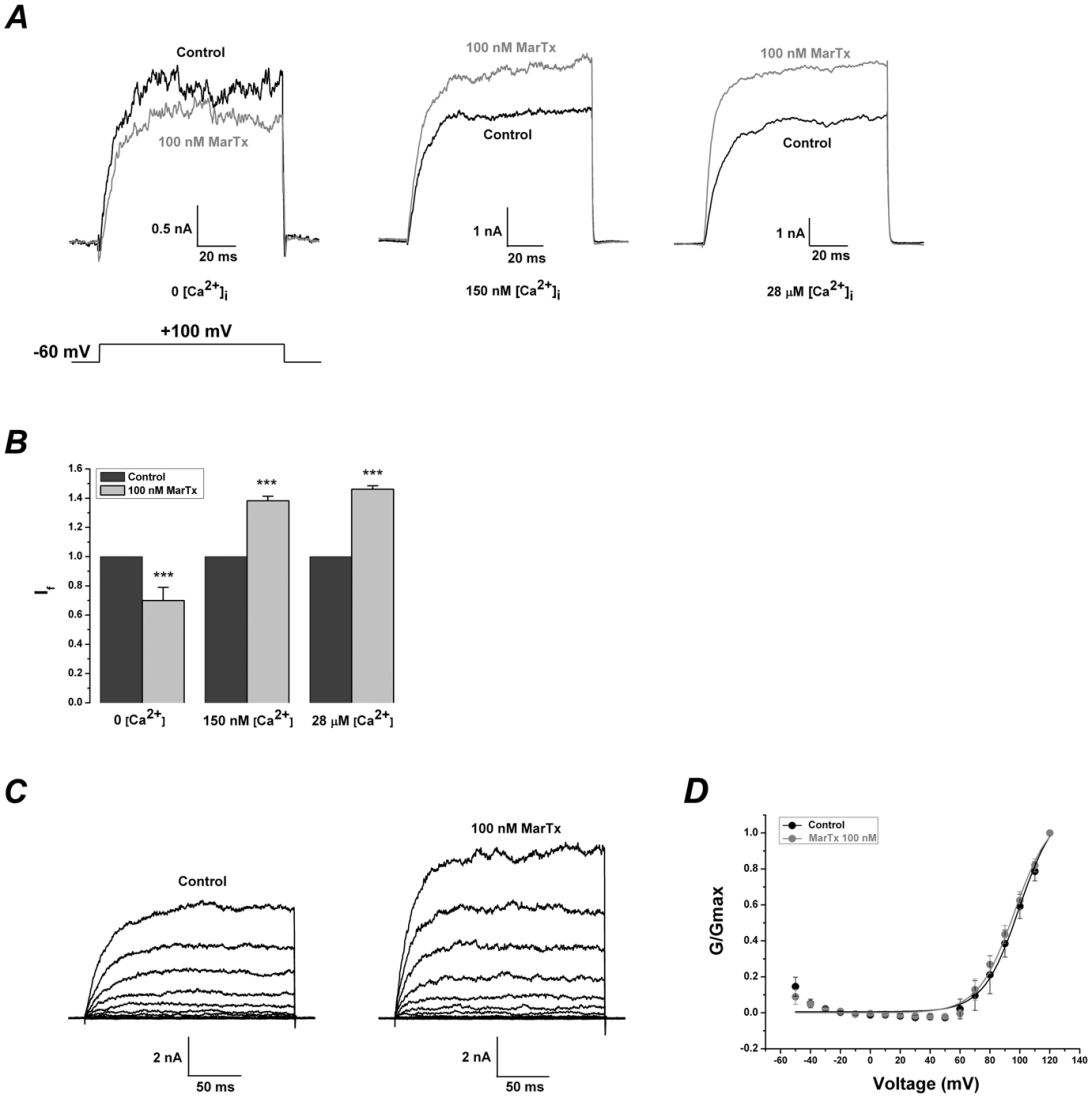
Ca^2+^-dependent and voltage-independent modulation of martentoxin on gBK channels. (A) Representative whole cell current traces from U251 cells before and after the application of martentoxin 100 nM. The holding voltage was −60 mV and the currents were elicited by a pulse of +100 mV with 0, 150 nM or 28 µM free Ca^2+^ in the pipette solution. (B) Statistics analysis of modulation of gBK channels by martentoxin 100 nM in the presence of 0 (n = 4), 150 nM (n = 8), 28 µM (n = 3) free Ca^2+^ in the pipette solution, respectively. *** p<0.001. The enhancive ratios of martentoxin on the gBK currents were not significantly different between 150 nM or 28 µM free Ca^2+^ in the pipette solution (n  = 3, p>0.05). (C) Representative whole cell current traces from U251 cells before (left) and after (right) the application of martentoxin 100 nM. The holding voltage was −60 mV and the currents was elicited by the step pulses ranging from – 50 to +120 mV for 200 ms with the increments of 10 mV. (D) The plots of the normalized conductance as a function of command potential in the absence or presence of martentoxin, data points were fitted using the Boltzmann function (“see Data analysis”). The voltage-dependent activation curve was not significantly shifted in the presence of martentoxin (n = 5).

In the case that U-251 cells were pre-treated with 200 nM thapsigargin, an irreversible inhibitor of the sarcoplasmic reticulum Ca^2+^-ATPase pump for 30 min at 37°C in order to deplete intracellular Ca^2+^ stores before performing whole cell patch recordings of gBK currents[36]. The pipette solution contained 10 mM EGTA but without Ca^2+^, the gBK channels were blocked by 100 nM martentoxin (I_f_:0.70±0.09, n = 4). When the free Ca^2+^ concentration was elevated to 150 nM or 28 µM, the activities of gBK channels were conversely enhanced by 100 nM martentoxin. But the enhancement ratios of gBK channel activities were not significantly different (I_f_: 1.38±0.03,n = 8; 1.46±0.02, n = 4, p>0.05). (see Fig. 4A and 4B).

Ca^2+^ imaging showed that the cytoplasmic Ca^2+^ concentration in glioma cells could not be oscillated by 100 nM or 500 nM martentoxin. Contrary to this, an obvious Ca^2+^ rise could be evoked by the solution with 100 mM caffeine (Fig. S2A), indicating that the cytoplasmic Ca^2+^ concentration after the establishment of the whole-cell mode was consistent with that in the pipette solution and remained almost unchanged during the recording. Consequently, the enhancement of gBK channel activity was induced by martentoxin directly.

When the free Ca^2+^ concentration in the pipette solution was remained at 150 nM, the gBK currents were elicited by the step pulses ranging from – 50 to +120 mV for 200 ms with the increments of 10 mV. Thus, the toxin effects on the voltage dependence of steady-state activation were analyzed as described in “Data analysis”. By perfusion with 100 nM martentoxin, the activation curve of gBK channels as well as the half-maximal voltage (V_1/2_) of activation was not significantly shifted with the application of 100 nM martentoxin (99.5±2.94 compared to 96.3±2.64,n = 5, p>0.05) (Fig. 4D and Table 1).

**Table 1:** The voltage independence of activation of gBK and BK channel (α+β1) subtypes in the absence and presence of martentoxin.

	V_1/2_ (mV)	k (mV)	n
gBK			
control	99.50±2.94	12.01±1.66	5
100 nM MarTx	96.33±2.64, P>0.05	12.22±1.60, P>0.05	5
BK (α+β1)			
control	65.56±1.68	24.31±1.39	8
1 µM MarTx	68.52±3.03, P>0.05	28.29±2.39, P>0.05	8

The groups of control and MarTx have no significantly difference, at the level of 0.05, tested by one-way ANOVA.

### Dose-dependent and voltage-independent modulation of martentoxin on BK channel (α+β1) expressed in HEK293T cells

**Figure 5:**
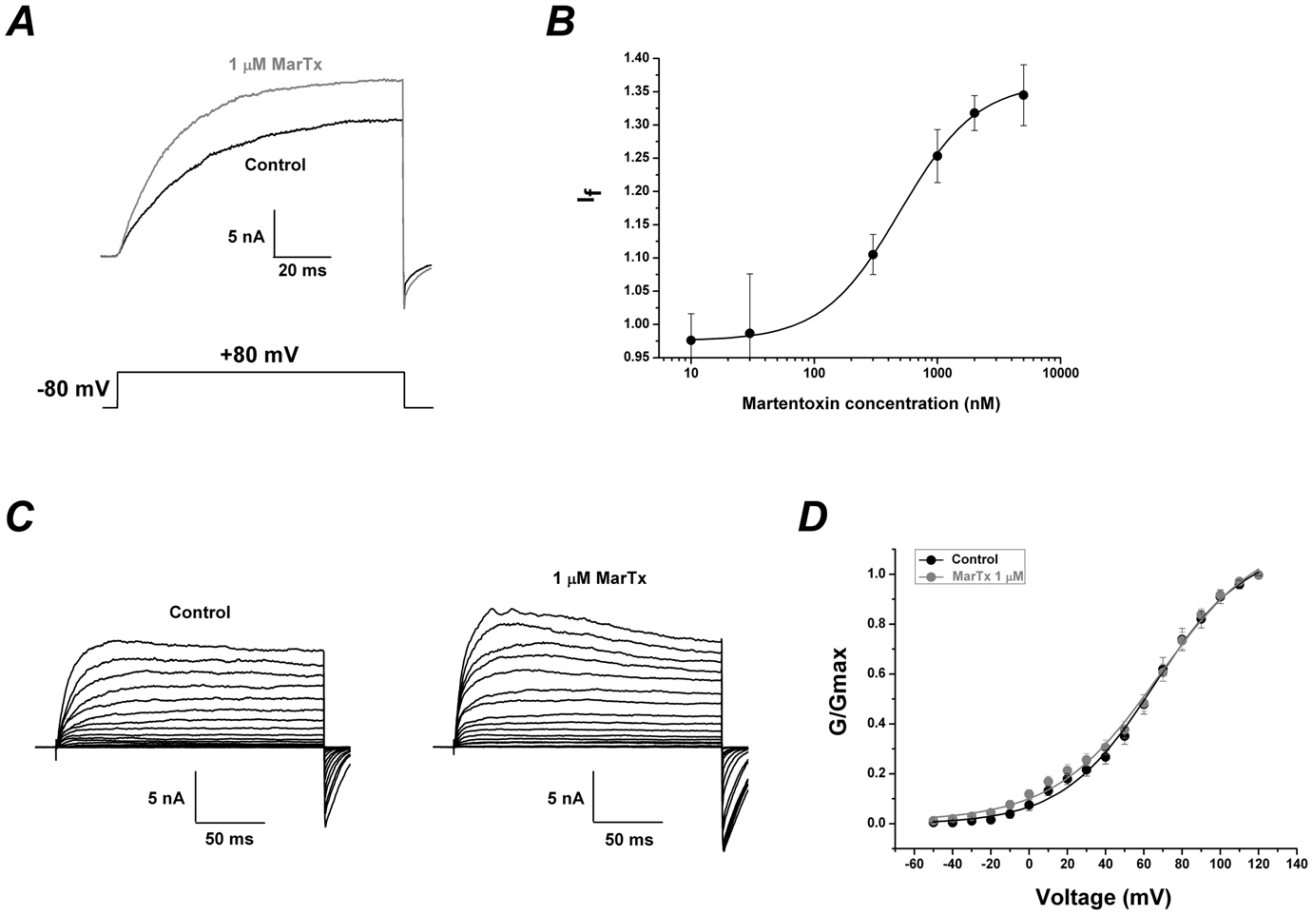
Dose-dependent and voltage-independent modulation of martentoxin on BK channels (α+β1) expressed in HEK293T cells. (A) Representative whole cell current traces from HEK 293T cells expressing BK channels (α+β1) before and after the application of martentoxin 1 µM. The holding voltage was −80 mV and the currents were elicited by a pulse of +80 mV with 700 nM free Ca^2+^ in the pipette solution. (B) The dose-response curve of martentoxin enhancing BK currents was fitted by the Hill equation (see “Data analysis”). The EC_50_ value is 495±26.7 nM, and the Hill coefficient is n = 1.39±0.09 (n = 3–5). (C) Representative whole cell current traces from HEK 293T cells expressing BK channels (α+β1) before (left) and after (right) the application of martentoxin 1 µM. The holding voltage was −80 mV and the currents were elicited by the step pulses ranging from – 50 to +120 mV for 200 ms with the increments of 10 mV. (D) The plots of the normalized conductance fit well with Boltzmann function (“see Data analysis”). The voltage-dependent activation curve was insignificantly shifted in the presence of martentoxin (n = 8).

In the case that free Ca^2+^ concentration in the pipette solution remained at 700 nM, the currents of BK channel (α+β1) were increased by martentoxin in the dose-dependent manner (Fig. 5A and 5B). The EC_50_ of martentoxin on BK channel (α+β1) was assessed to be 495±26.7 nM with a Hill coefficient of n = 1.39±0.09 according to the dose-response curve fitting (n = 3–5). In addition, the effects of martentoxin on steady-state activation of α+β1 subtype was studied by the same experimental voltage protocol and the same data analysis as gBK channels (see “Data analysis”). A similar effect could be observed in the presence of 1 µM martentoxin. The activation curve of α+β1 BK channels and the half-maximal voltage (V_1/2_) of activation were also insignificantly moved (65.5±1.68 compared to 68.5±3.03, n = 8, p>0.05) (Fig. 5D and Table 1).

### Ca^2+^-dependent modulation of martentoxin on BK channel (α+β1) expressed in HEK293T cells

**Figure 6:**
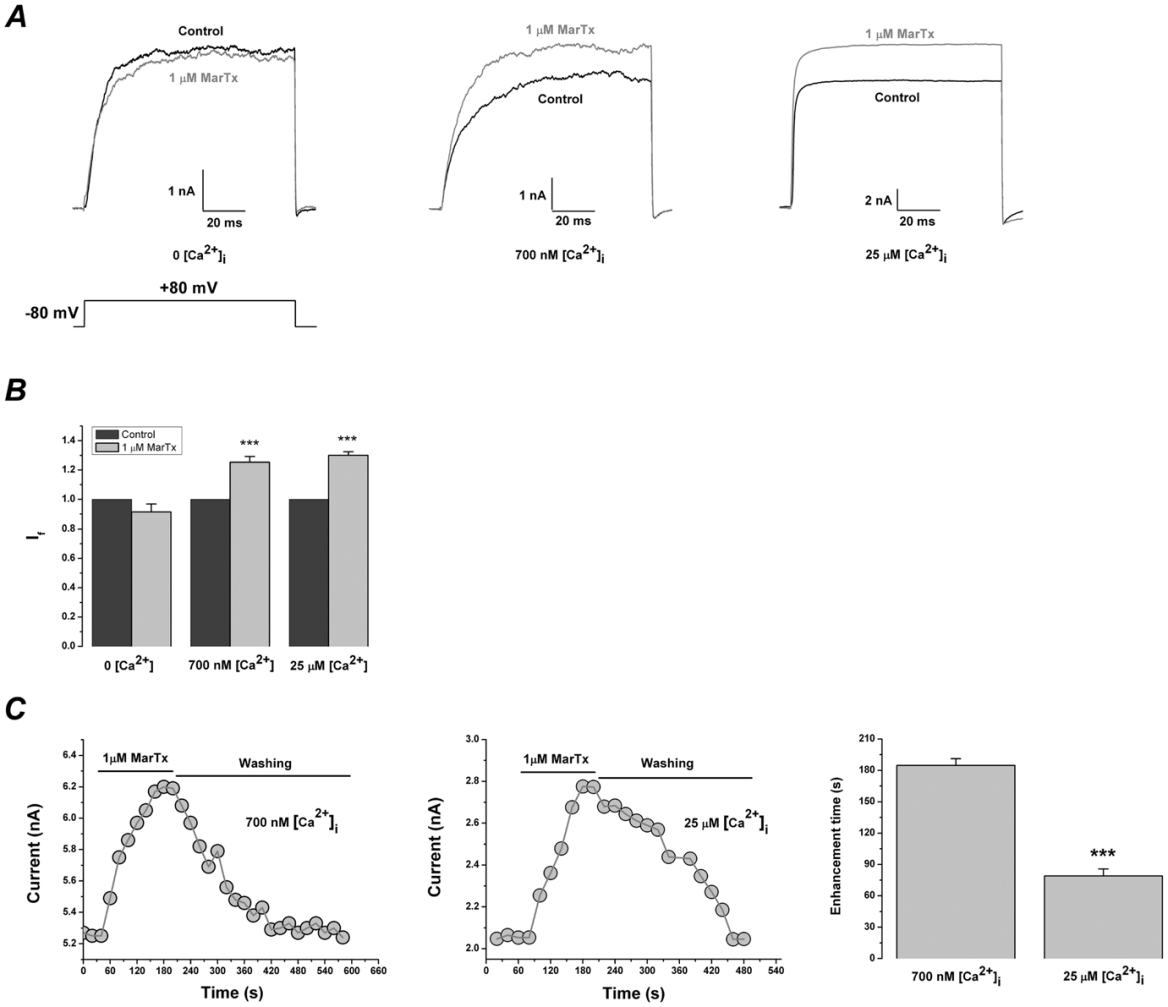
Ca^2+^-dependent modulation of martentoxin on BK channels (α+β1) expressed in HEK293T cells. (A) Representative whole cell current traces from HEK 293T cells expressing BK channels (α+β1) before and after the application of martentoxin 1 µM. The holding voltage was −80 mV and the currents were elicited by a pulse of +80 mV with 0, 700 nM or 25 µM free Ca^2+^ in the pipette solution. (B) Statistics analysis of modulation of BK channels (α+β1) by martentoxin 1 µM in the presence of 700 nM or 25 µM free Ca^2+^ in the pipette solution (n = 6). *** p<0.001. The enhancive ratios of martentoxin on the currents of α+β1 subtype were not significantly different between 700 nM or 25 µM free Ca^2+^ in the pipette solution (n = 4; p>0.05) (C) Time course for the up-modulation of BK channels (α+β1) by martentoxin 1 µM in the presence of 700 nM or 25 µM free Ca^2+^ in the pipette solution (left). Statistics analysis (n = 6) of the shortest time between the control current and the peak currents induced by martentoxin 1 µM (right). p>0.05.

In the case that HEK293T cells transfected with BK channel (α+β1) were also pre-treated with 200 nM thapsigargin for 30 min at 37°C. When Ca^2+^ was fully excluded from the pipette solution, the currents of BK channel (α+β1) evoked by +80 mV pulse could not be modulated by martentoxin even at 1 µM (Fig. 6A). However, when free Ca^2+^ in the pipette solution was remained at 700 nM or 25 µM, the currents of BK channel (α+β1) were enhanced by martentoxin 1 µM (Fig. 6A). Similar to the modulation of martentoxin to gBK channel, the enhancive ratios of martentoxin on the currents of BK channel (α+β1) were not significantly different those at 700 nM or 25 µM free Ca^2+^ in the pipette solution (I_f_: 1.25±0.04,n = 4 compared to 1.30±0.02, n = 4; p>0.05) as shown in Fig. 6B. Nevertheless, the least time from the control current to the peak current after the application of martentoxin was distinguishing between 700 nM and 25 µM free Ca^2+^ in the pipette solution. The shortest time was 185±6.4 s (n = 6) at 700 nM free Ca^2+^, but shortened to 79.0±6.7 s (n = 6) at 25 µM free Ca^2+^ (Fig. 6C).

Ca^2+^ imaging showed that the cytoplasmic Ca^2+^ concentration in HEK 293T cells could not be oscillated by 1 µM martentoxin, suggesting that the cytoplasmic Ca^2+^ concentration remained almost unchanged during the recording (Fig. S2B). Therefore, it is clear that the martentoxin could modulate BK channel (α+β1) directly, without elevating cytosolic Ca^2+^.

### Iberiotoxin abolished the enhancement of martentoxin on the activity of BK channel (α+β1)

**Figure 7:**
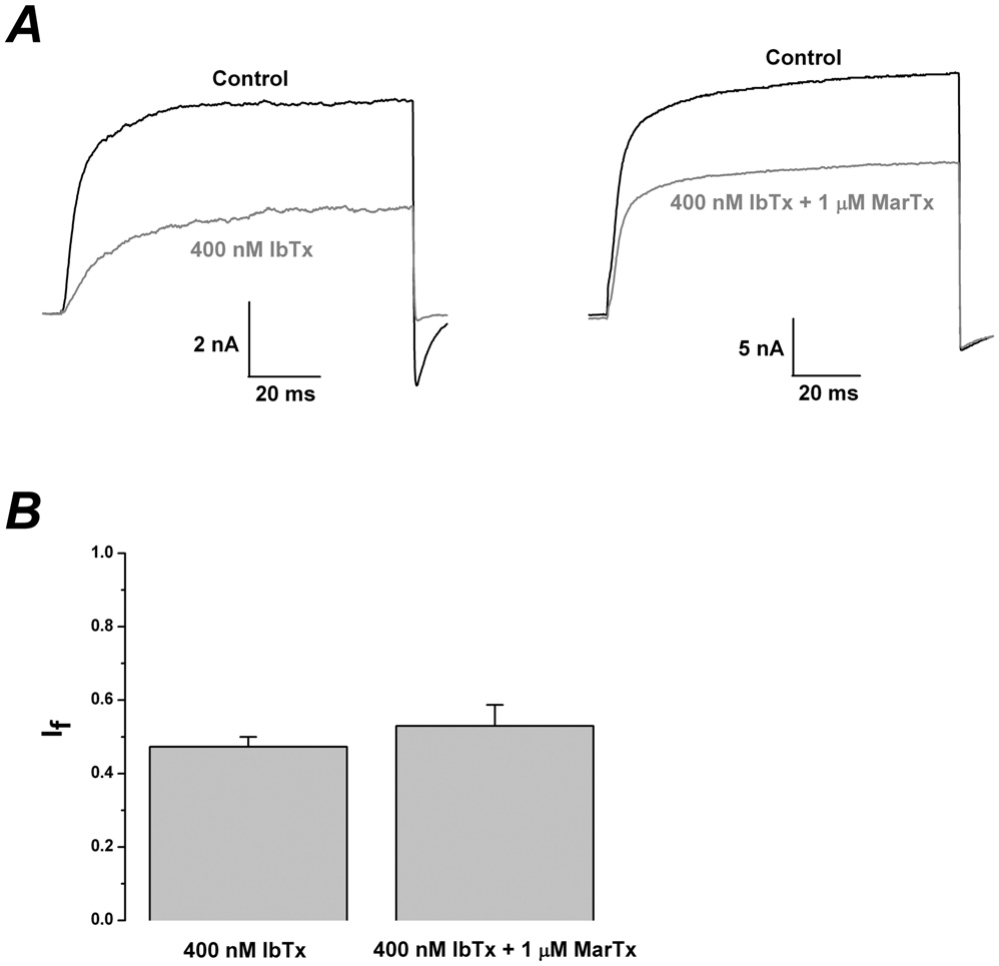
The enhancement effect of martentoxin on BK channels (α+β1) could be fully abolished by IbTx. (A) Representative whole cell current traces from HEK 293T cells expressing BK channels (α+β1) before and after the application of IbTx 400 nM (left) or the mixture of IbTx 400 nM and martentoxin 1 µM (right). The holding voltage was −80 mV and the currents were elicited by a pulse of +80 mV in the presence of 700 nM free Ca^2+^ in the pipette solution (Fig. 5A). (B) Statistics analysis of pharmacological modulation of gBK channels by IbTx 400 nM (n = 5) or the mixture of IbTx 400 nM and martentoxin 1 µM. (n = 4) p>0.05.

The currents of BK channel (α+β1) could be markedly inhibited by iberiotoxin, a pore blocker of BK channels, or the mixture of iberiotoxin and martentoxin. I_f_ was 0.47±0.03 (n = 5) at 400 nM iberiotoxin. Similarly, I_f_ was 0.53±0.06 (n = 4) with the mixture of 1 µM martentoxin and 400 nM iberiotoxin (Fig. 7).

## Discussion

The modulatory characteristics of gBK channel and BK channel (α+β1), two Ca^2+^-hypersensitive BK channel subtypes, by martentoxin were investigated in the present study. The activities of both BK channel (α+β1) and gBK channel could be enhanced by martentoxin, depending on the presence of cytoplasmic free Ca^2+^.

### Selectivity of martentoxin on BK channel subtypes

BK channels consist of two distinct subunits, the pore-forming α subunit (*slo*) and the β regulatory subunit [37]. Alternative splicing of the *slo* mRNA [27] as well as tissue-specific β subunits are found to give rise to diverse subtypes [38]. However, other specific modulators for BK channel have not been found to effectively discriminate these subtypes.

Our initial study showed that 100 nM martentoxin could potently block BK channel currents in adrenal medulla chromaffin cells in which β2 subunit may be coexpressed with α subunit [31]. Subsequently, it was shown that the iberiotoxin-insensitive neuronal BK channels (α+β4) could be strongly blocked by martentoxin, while the iberiotoxin-sensitive BK channel consisting of only α subunit was almost insensitive to martentoxin [34]. In the present study, it was found that the activities of gBK and BK channel (α+β1) subtypes were both enhanced by martentoxin. Furthermore, martentoxin displayed a higher preference with gBK channel over BK channel (α+β1) by about 10 folds (Fig. 3B and 5B). This subtype-selectivity of martentoxin may possibly attribute to the different modulatory effects of diverse β subunits or alternative splicing of α subunit of BK channels. Over all, martentoxin could be utilized not only as a specific blocker for neuronal BK channel subtype but also as a modulator for discriminating most BK channel subtypes.

### Ca^2+^-dependent modulation of martentoxin on BK channel subtypes

Ca^2+^ sensor of the BK channel formed by only α subunits is composed of multiple Ca^2+^ binding sites distributing in the cytoplasmic C-terminal region. These sites bind Ca^2+^ with different affinity[39]. Since the Ca^2+^ or voltage sensor for BK channel activation can act relatively independently to each other [40], [41], it is thus implied that the activation conformation of BK channel induced by variant cytoplasmic Ca^2+^ concentrations may be different.

Our previous study showed that the neuronal BK channel (α+β4) currents could be reduced by martentoxin in the presence of low cytoplasmic Ca^2+^ concentration, but conversely increased in the presence of high cytoplasmic Ca^2+^ concentration[34]. That is corresponding to the notion that β4 subunit reduces BK channel openings at low cytoplasmic Ca^2+^ while increases channel openings at high cytoplasmic Ca^2+^ [42]. It is thus indicated that the pharmacological effects of martentoxin would be reversed subject to the change of conformation of BK channel.

In this study, it was found that the activities of gBK and BK channel (α+β1) subtypes were both up-modulated by martentoxin only in the presence of cytoplasmic free Ca^2+^. When Ca^2+^ was completely removed from the pipette solution, the activity of gBK channel was conversely inhibited, but the activity of BK channel (α+β1) was unaltered by martentoxin. It thus clearly suggested whether the presence or absence of cytoplasmic Ca^2+^ was also crucial to the effects of martentoxin on such BK channel subtypes.

BK channel possesses many subtypes and it still remains unknown how martentoxin modulates other BK channel subtypes. But the present results implied that the pharmacological effects of martentoxin on BK channel subtypes depended partly on the Ca^2+^ binding sites of BK channels directly or indirectly. Since there is a close coupling between Ca^2+^ binding site and other regions especially β subunit of BK channel, martentoxin may bind with these specific regions correlated closely with Ca^2+^ binding sites because as a peptide, martentoxin could not reach the cytoplasm of the cell.

### Up-modulatory mechanism of martentoxin on activity of gBK and BK channel (α+β1) subtypes

gBK channels are abundant in human glioma cells and contribute to ∼90% of the outward currents. This study found that, contrary to iberiotoxin[17], martentoxin could accelerate the proliferation of glioma cells (Fig. 3C and D). It strongly suggested that gBK channel was the direct target of martentoxin. Moreover, the block potency on the outward currents with the simultaneous application of martentoxin and iberiotoxin was not significantly different from that by iberiotoxin alone. The result indicated that i) the remaining NPPB sensitive Cl^−^ channel was not recognized by martentoxin, ii) the receptor site of gBK channel associating with martentoxin and iberiotoxin was not overlapping. As the pore region was usually regarded as the target of iberiotoxin, the docking sites of gBK channel for martentoxin might be far from the pore region. The voltage-dependence of gBK and BK channel (α+β1) was not shifted by martentoxin (Fig. 4D and 5D), which rules out the possibility that martentoxin interacts directly with the voltage sensor. Furthemore, accumulated data supported that β subunit might affect the pharmacological or Ca^2+^ sensitive characteristics of gBK channel[25], [27]. It allow us to speculate that β subunit of gBK channel might underlie the receptor sites for martentoxin. Similarly, BK channel consisted of α subunit alone was insensitive to martentoxin even at the concentration of toxin elevated to 1 µM (Fig. S3). Moreover, the enhancement of martentoxin on the activity of BK channel (α+β1) could be completely abolished by IbTx (Fig. 7). What's more, our recent preliminary work showed that the extracellular loop of β1 subunit plays a crucial role in interacting with martentoxin (unpublished data Tao et al.). Hence, these results strongly implied that the enhancement of martentoxin on these BK channel subtypes may come from its binding directly to β1 subunit.

### Martentoxin, a valuable tool to BK channel-related diseases research and drug-design

Malignant gliomas are the most common primary intracranial tumors with high mortality [43]. Prominent expression of BK channel in human glioma cells is correlated positively with enhanced malignancy grades [27], [28]. Our results showed that martentoxin displayed a high sensitivity on glioma BK channels (Fig. 3B). It was strongly implied that martentoxin, a molecular probe of gBK channel, could be used for testing human gliomas. Moreover, proliferation of U251 cells could be obviously enhanced by martentoxin under the serum-free condition (Fig. 3C and 3D). The underlying mechanism of gBK channels affecting on the cell cycle remains unknown. Also, it is still unclear whether martentoxin could protect normal glia cells under the serum-free condition. Such investigations with martentoxin may clarify the mechanism how the gBK channel produce or relate to the malignant proliferation.

On the other hand, BK channel (α+β1) activated by local Ca^2+^ release could regulate the membrane potential of arterial smooth muscle cells and protect against hypertension [6]. Activation of BK (α+β1) channels by pharmacologic tools may be an effective treatment for hypertension disorders with increased smooth muscle tone. This study showed that the currents of BK channel (α+β1) could be strongly increased by martentoxin (Fig. 5A and 5B). It may allow us to speculate that martentoxin could be utilized as a scaffold for designing novel modifiers to enhance channel activity.

## Supporting Information

Figure S1 **Sensitivity of the remaining iberiotoxin-insensitive outward currents to NPPB in U251 cells.** (A) Representative whole cell current traces from U251 cells before and after the single application of iberiotoxin (IbTx) 100 nM, and simultaneous application of iberiotoxin 100 nM and NPPB 200 µM. The holding voltage was −60 mV and the currents were elicited by a pulse of +100 mV (see Fig. 1A). (B) Statistics analysis of unblocked current (I_f_) after iberiotoxin 100 nM (n = 6) and the mixture of iberiotoxin and NPPB (n = 6). P<0.001.
(TIF)

Figure S2 **Effects of martentoxin on cytoplasmic Ca^2+^.** (A) Effects of martentoxin on cytoplasmic Ca^2+^ of U251 cells. After the application of martentoxin 100 nM or 500 nM, the cytoplasmic Ca^2+^ concentration was unchanged. (B)Effects of martentoxin on cytoplasmic Ca^2+^ of HEK 293T cells. After the application of martentoxin 1 µM, the cytoplasmic Ca^2+^ concentration was unchanged.
(TIF)

Figure S3 **Slight effect of martentoxin on BK channels (hsloα alone).** (A) Representative current traces are shown. The channels were activated by +80 mV with a −80 mV holding potential (see Fig. 5A). The free Ca^2+^ concentration in the pipette solution was 700 nM. The currents were hardly altered by 1 µM of martentoxin. (B) The time course curve confirmed the lack of sensitivity of this type (α alone) of BK channels to martentoxin.
(TIF)
