# Supplementary figures and images for: Enhancement Effects of Martentoxin on Glioma BK Channel and BK Channel (α+β1) Subtypes
Source: PLoS One. 2011 Mar 18;6(3):e15896. doi: 10.1371/journal.pone.0015896 (PMC3060806; doi:10.1371/journal.pone.0015896)

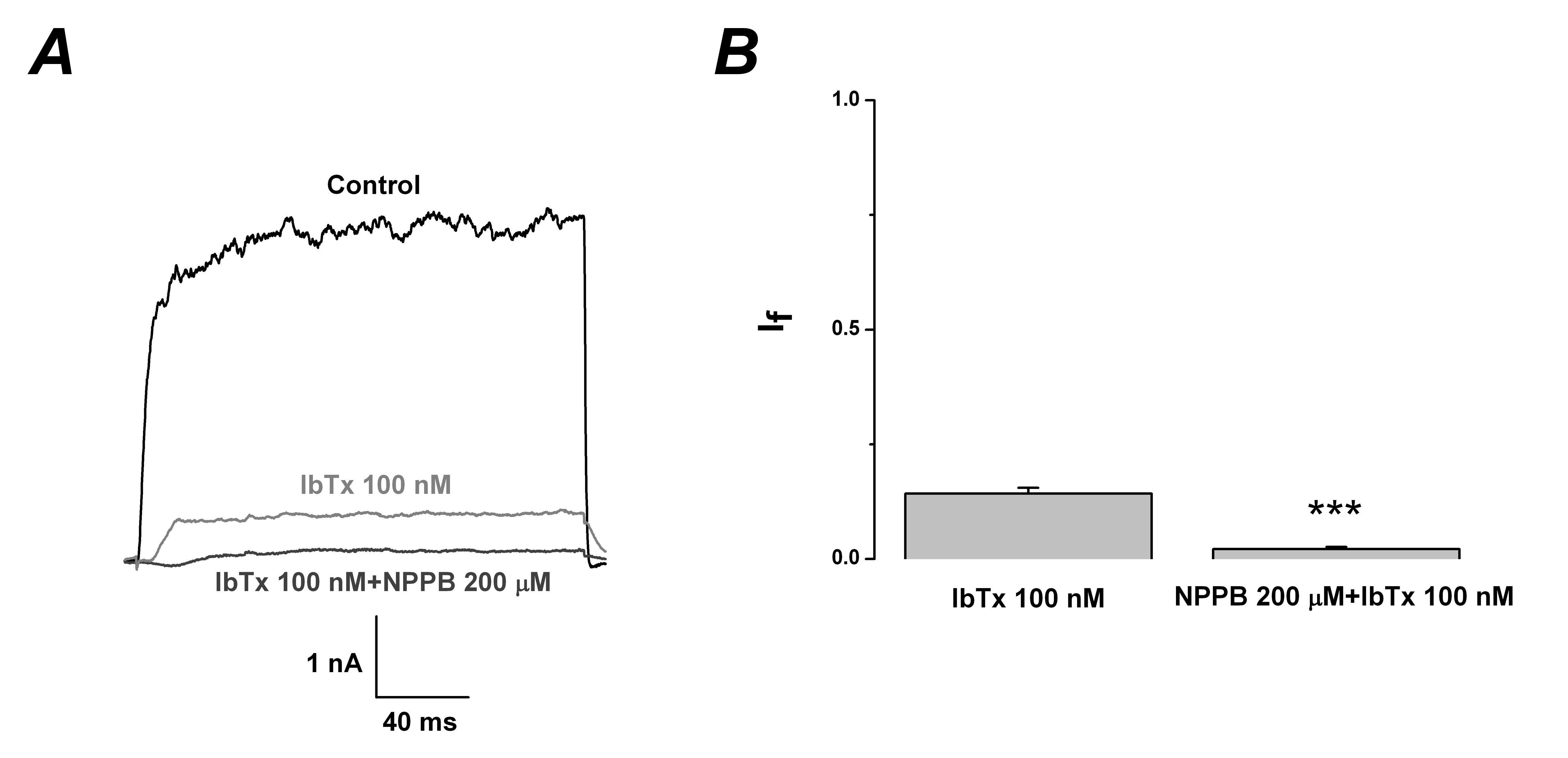

Supplement: Figure S1 — Sensitivity of the remaining iberiotoxin-insensitive outward currents to NPPB in U251 cells. (A) Representative whole cell current traces from U251 cells before and after the single application of iberiotoxin (IbTx) 100 nM, and simultaneous application of iberiotoxin 100 nM and NPPB 200 µM. The holding voltage was −60 mV and the currents were elicited by a pulse of +100 mV (see Fig. 1A). (B) Statistics analysis of unblocked current (If) after iberiotoxin 100 nM (n = 6) and the mixture of iberiotoxin and NPPB (n = 6). P<0.001. (TIF) [file pone.0015896.s001.tif]

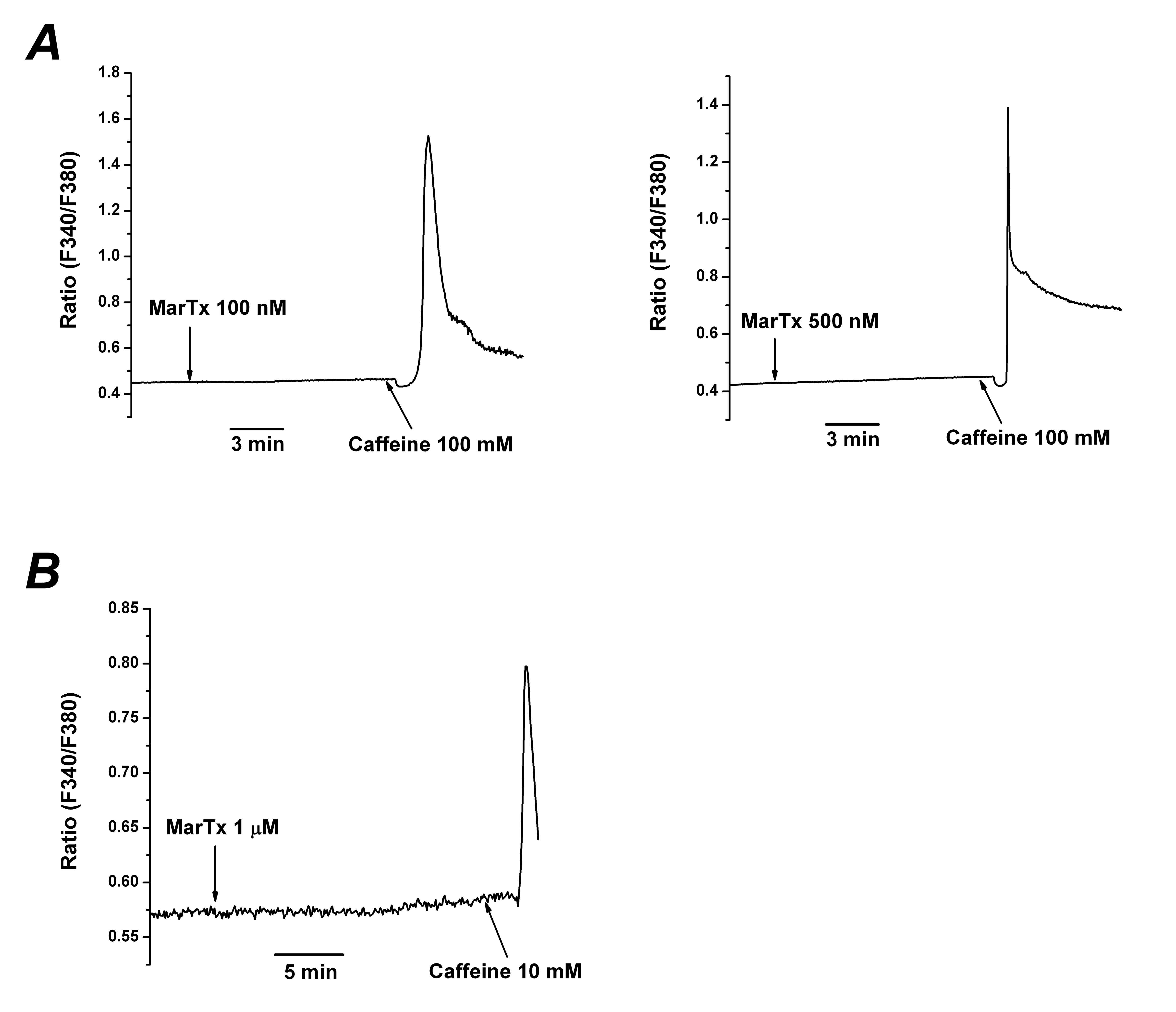

Supplement: Figure S2 — Effects of martentoxin on cytoplasmic Ca2+. (A) Effects of martentoxin on cytoplasmic Ca2+ of U251 cells. After the application of martentoxin 100 nM or 500 nM, the cytoplasmic Ca2+ concentration was unchanged. (B)Effects of martentoxin on cytoplasmic Ca2+ of HEK 293T cells. After the application of martentoxin 1 µM, the cytoplasmic Ca2+ concentration was unchanged. (TIF) [file pone.0015896.s002.tif]

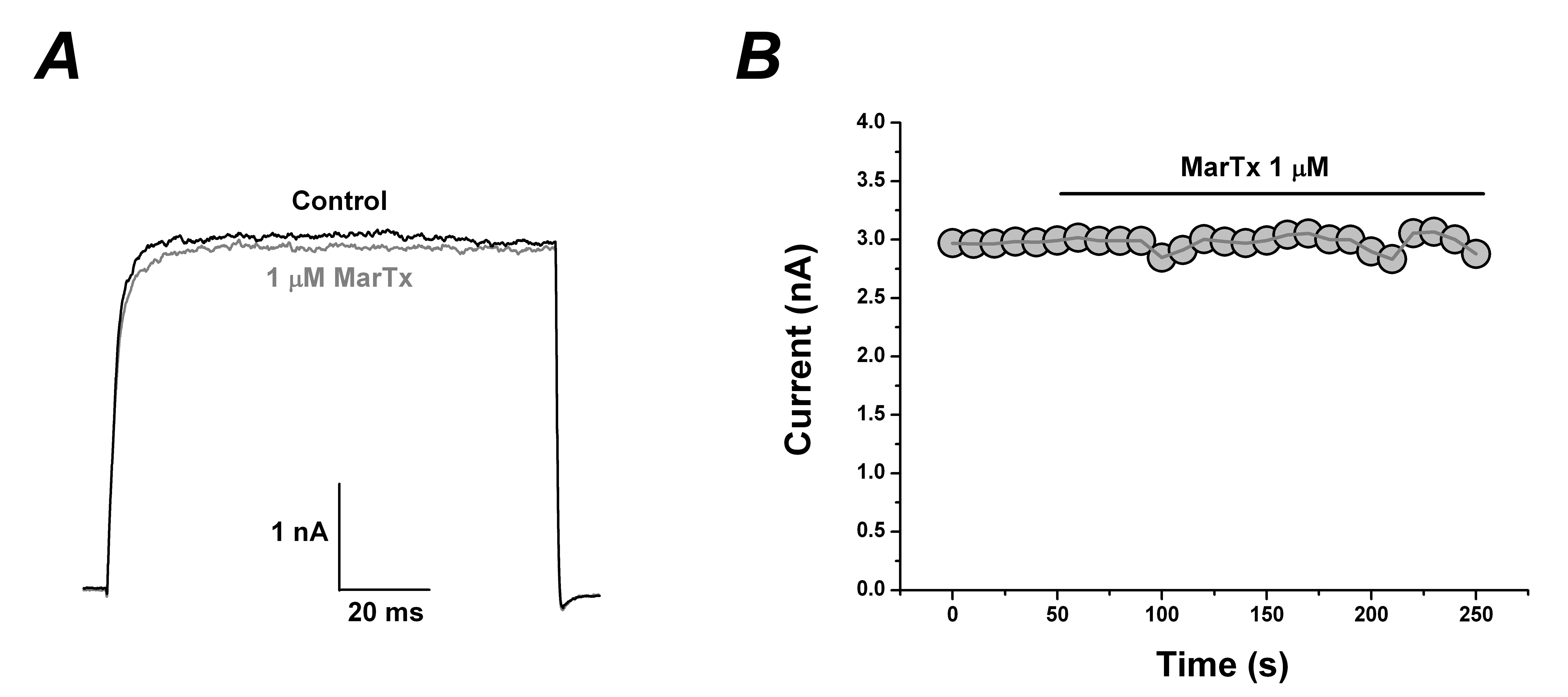

Supplement: Figure S3 — Slight effect of martentoxin on BK channels (hsloα alone). (A) Representative current traces are shown. The channels were activated by +80 mV with a −80 mV holding potential (see Fig. 5A). The free Ca2+ concentration in the pipette solution was 700 nM. The currents were hardly altered by 1 µM of martentoxin. (B) The time course curve confirmed the lack of sensitivity of this type (α alone) of BK channels to martentoxin. (TIF) [file pone.0015896.s003.tif]
